# *Aquilaria sinensis* leaf tea affects the immune system and increases sleep in zebrafish

**DOI:** 10.3389/fphar.2023.1246761

**Published:** 2023-11-16

**Authors:** Xiaohui Tan, Liping Wang, William Kojo Smith, Huayan Sun, Lingyun Long, Liyan Mao, Qiuwei Huang, Huifang Huang, Zhaomin Zhong

**Affiliations:** ^1^ School of Biology and Basic Medical Sciences, Soochow University, Suzhou, China; ^2^ Guangxi Subtropical Crops Research Institute, Nanning, China; ^3^ Key Laboratory of Quality and Safety Control for Subtropical Fruit and Vegetable, Ministry of Agriculture and Rural Affairs, Nanning, China

**Keywords:** light exposure, sleep disturbance, *Aquilaria sinensis*, zebrafish, immune system

## Abstract

The importance of adequate sleep for good health cannot be overstated. Excessive light exposure at night disrupts sleep, therefore, it is important to find more healthy drinks that can promote sleep under sleep-disturbed conditions. The present study investigated the use of *A. sinensis* (Lour.) Spreng leaf tea, a natural product, to reduce the adverse effects of nighttime light on sleep. Here, *Aquilaria sinensis* leaf tea at 1.0 and 1.5 g/L significantly increased sleep time in zebrafish larvae (5–7 dpf) with light-induced sleep disturbance. Transcriptome sequencing and qRT-PCR analysis revealed a decrease in the immune-related genes, such as *nfkbiab*, *tnfrsf1a*, *nfkbiaa*, *il1b*, *traf3*, and *cd40 in the* 1.5 g/L *Aquilaria sinensis* leaf tea treatment group. In addition, a gene associated with sleep, *bhlhe41*, showed a significant decrease. Moreover, *Aquilaria sinensis* leaf tea suppressed the increase in neutrophils of *Tg(mpo:GFP)* zebrafish under sleep-disturbed conditions, indicating its ability to improve the immune response. Widely targeted metabolic profiling of the *Aquilaria sinensis* tea using ultra-performance liquid chromatography coupled with electrospray tandem mass spectrometry (UPLC-ESI-MS/MS) revealed flavonoids as the predominant component. Network pharmacological and molecular docking analyses suggested that the flavonoids quercetin and eupatilin in *Aquilaria sinensis* leaf tea improved the sleep of zebrafish by interacting with *il1b* and *cd40* genes under light exposure at night. Therefore, the results of the study provide evidence supporting the notion that *Aquilaria sinensis* leaf tea has a positive impact on sleep patterns in zebrafish subjected to disrupted sleep due to nighttime light exposure. This suggests that the utilization of *Aquilaria sinensis* leaf tea as a potential therapeutic intervention for sleep disturbances induced by light may yield advantageous outcomes.

## 1 Introduction

*Aquilaria sinensis* (Lour.) Spreng, or Agarwood is a traditional spice and valuable Chinese medicine. It belongs to the Thymelaeaceae tree family. For religious and medicinal purposes, *Aquilaria sinensis* resin and oil are used in perfumes and incense (Naziz et al., 2019). Traditional medicine uses *A. sinensis* resin or oil to treat inflammation, diarrhea, joint pain, cardiovascular disease, insomnia, and stimulate the body (Pang et al., 2023a). *A. sinensis* leaves are secondary agricultural products before the wood can be harvested. There are many health benefits associated with *A. sinensis* leaves, and traditionally, these leaves have been consumed as tea, especially in Asia and Southeast Asia (Han and Li, 2012; Adam et al., 2017; Surjanto et al., 2019). Studying the biological activity of *A. sinensis* leaf extract will help expand the plant’s therapeutic and medicinal potential and realize the comprehensive utilization of resources. The extracts from agarwood leaves exhibit a variety of biological activities, including antimicrobial, anticancer, and anti-inflammatory properties (Cheng et al., 2013; Kamonwannasit et al., 2013; Eissa et al., 2021). Additionally, *A. sinensis* leaf extracts may help improve the immune response of macrophages by regulating pro-inflammatory cytokines and also enhance innate immunity against infection on RAW264.7 murine macrophages. Investigating these aspects using animal models will validate.

Sleep is a physiological phenomenon that has been hypothesized to possess rejuvenating and regulatory attributes (Benington and Craig Heller, 1995; Mackiewicz et al., 2007). Despite the lack of precise understanding regarding its specific purpose, sleep has garnered significant attention in recent times owing to its potential impact on the immune system. Numerous investigations have provided evidence that sleep disturbance alters diverse elements of the immune system, including the proportions of cell subpopulations (such as CD4^+^ and NK) and levels of cytokines (such as IFN-g, TNF-a, and IL-1) (Dimitrov et al., 2007; Zager et al., 2007; Yehuda et al., 2009). Earlier we mentioned that *A. sinensis* leaf extracts may help improve the immune response, so this study aims to investigate the effect of tea on sleep.

Zebrafish is one of the best model organisms used in research due to its tremendously large spawning capacity, *in vitro* development, transparent embryos, and highly homologous to humans. Exposing zebrafish larvae to chemical treatments is considerably simpler and easier than exposing mammals. Thus, it is an ideal vertebrate model for evaluating the efficacy of natural products, food, and drugs and studying sleep and immune responses (Prober et al., 2006; Appelbaum et al., 2009; Rihel et al., 2010; Varela et al., 2017; Tran and Prober, 2022). Furthermore, akin to humans, zebrafish exhibit a comparable sleep-wake cycle, and the presence of nighttime illumination can greatly impede zebrafish sleep, resulting in sleep disturbances (Yokogawa et al., 2007; Hirayama et al., 2019). In this study, to investigate the new efficacy of *A. sinensis* leaf tea, using behavior, transcriptomics, metabolomics, and network pharmacology, we found the improved effects of sleep-disturbed and immune functions of *A. sinensis* leaf tea in zebrafish.

## 2 Material and methods

### 2.1 Zebrafish culture

Adults of AB and *Tg(mpo:GFP)* (Renshaw et al., 2006) zebrafish (*Danio rerio*) lines were cultured in a circulating water system under a 14 h/10 h light-dark cycle and a constant temperature of 28°C; these adults were fed three times per day. The male and female zebrafish were paired at night, and eggs were laid the following day within 1 h after turning on the lights. These embryos were placed in 10 cm dishes containing 1 × E3 medium (The pH was adjusted to 7.0–7.2 using 5 mM NaCl, 0.17 mM KCl, 0.33 mM CaCl_2_ and 0.33 mM MgSO_4_) with methylene blue (0.3 ppm) and reared in a light-controlled incubator (14 h/10 h light-dark cycle, ZT 0–ZT 14, light condition; ZT 14–ZT 24, dark condition; ZT: Zeitgeber Time) at 28°C. All experimental procedures of this study were approved by the Animal Care and Use Committee of Soochow University and performed following Chinese government regulations.

### 2.2 Boiling water extraction of *A. sinensis* leaf tea

*Aquilaria sinensis* leaf tea was from commercially available *A. sinensis* leaf tea (Yishen Tea Co., Ltd. Lianjiang City, China) and processed following the regional standard “technical specification” for the production of organic oolong tea in Guangdong Province (https://www.sdtdata.com/fx/fmoa/tsLibCard.doView). Then, the guidelines outlined in the “Technical Guidelines for Toxicological Examination and Evaluation of Health Food and Its Raw Materials Safety (2020 edition)" https://www.samr.gov.cn/tssps/tzgg/zjwh/202010/t20201031_322804.html) were followed to obtain the leaf extract. Here, the *A. sinensis* leaf tea were weighed, then put into a 50 mL conical flask, and 25 mL of 1 × E3 medium (100°C) was added; after 30 min, the extract was transferred to a new 50 mL flask from the conical flask, this process was repeated twice and the extracts combined. The extract was then centrifuged at 1000 rpm and 28°C for 5 min, and the supernatant obtained was used as the leaf tea for subsequent experiments.

### 2.3 Determination of semi-lethal concentration of *A. sinensis* leaf tea in zebrafish

Zebrafish larvae at 5 dpf (days post fertilization) were housed in 24-well plates (ten fish per well) containing 2 mL of maintaining a well without tea as the negative control and three replicate wells per concentration under 14 h/10 h light-dark cycle and 28°C conditions. The number of dead zebrafish larvae was recorded during daily processing and LC_50_ was calculated using Probit (assumes normal distribution) analysis (Finney, 1971) by GraphPad (version 8.0).

### 2.4 Effects of *A. sinensis* leaf tea, eupatilin, and quercetin on the sleep patterns of zebrafish with light-induced sleep disturbance

One zebrafish larva (5 dpf) was placed in each well of a 48-well plate and treated with 1 mL of *A. sinensis* leaf tea (0.5, 0.75, 1.00, and 1.50 g/L), eupatilin (2, 4, 10 μM) **(**TargetMol, Boston, MA, United States), and quercetin (40, 80, 160 μM) (Shanghai Yuanye Bio-Technology CO., Ltd.) at varying concentrations; eight wells were maintained per treatment concentration. Sleep disturbance was induced by providing 300 lux illumination every night from 23:00–03:00, using a zebrafish behavior trajectory tracking system (ZebraBox, ViewPoint, France) which also recorded the behavior based on light exposure, and the sleep data were recorded using ZebraLab software (Version 2.3.1, ViewPoint, France) with the following parameters: detection threshold, 20; burst, 25; freeze, 4; bin size, 60 s. Then, the sleep and wake behaviors of each zebrafish were analyzed using MATLAB (Version R2021a) scripts as described in David Prober’s lab (Lee et al., 2017).

### 2.5 Transcriptome sequencing and data analysis

Thirty zebrafish larvae (5 dpf) were placed in each well of a six-well plate and treated with 3 mL of 1.5 g/L *A. sinensis* leaf tea, maintaining wells without the tea as a control and three wells per treatment as replicates. The zebrafish larvae were subjected to sleep disturbance by exposure to 300 lux light and collected at ZT 18 on the third day after treatment. RNA was extracted from these larval samples via Trizol reagent (Invitrogen, United States). Transcriptome sequencing was performed by BioMarker (Biomarker Technologies Corporation, Beijing, China), and the KEGG (Kyoto Encyclopedia of Genes and Genomes), COG (Clusters of Orthologous Groups of proteins), Ortholog database, and GO (Gene Ontology) database. The reads from each sample were assembled using the StringTie software, and the computation of FPKM (Fragments per kilobase of transcript per million mapped reads) was performed by both StringTie and edgeR in order to evaluate the expression levels of all mRNAs. Subsequently, the mRNAs and genes exhibiting differential expression levels were identified using the R package, with a criterion of log2 (fold change) > 1.2 or < −1.2 and a significance level of *p* < 0.05. The Biomarker cloud platform (Biomarker Biotechnology Co., Beijing, China) was used to prepare the transcriptome sequencing data statistics and a few tables.

### 2.6 Quantitative real-time PCR

As described previously, total RNA was extracted using TRIzol Reagent^®^ (Invitrogen), and cDNA was prepared by reverse transcription of 3 μg of DNAse I-treated total RNA. Then, qRT-PCR was performed using the cDNA on a StepOnePlus ABI instrument using SYBR green detection kit (Invitrogen) and the following thermal profile: 40 cycles of 10 s at 95°C and 30 s at 60°C. The relative expression levels of the *nfkbiab*, *cd40*, *traf3*, *il1b*, *nfkbiaa*, *tnfrsf1a*, *bhlhe41* were calculated following the 2^−△△^CT method (VanGuilder et al., 2008). The list of primers used in this study is shown in Supplementary Table S1.

### 2.7 Sleep disturbance and images of neutrophil-specific *Tg(mpo:GFP)* zebrafish

Ten *Tg(mpo:GFP)* larvae (5 dpf) were placed in each well of a 12-well plate in embryo culture medium with and without *A. sinensis* leaf tea (1.5 g/L). Light-induced sleep disturbance experiments were carried out on the zebrafish behavior trajectory tracking system (ZebraBox, ViewPoint, France) by providing 300 lux illumination every night from ZT 14 to ZT 18. On the third day of sleep disturbance, *Tg(mpo:GFP)* larvae were anesthetized at ZT 17–ZT 18, and images were captured after being treated with a fluorescence microscope (Axio Imager M2, Zeiss).

### 2.8 Widely targeted metabolomic profiling

The chemical constituents of 1.5 g/L *A. sinensis* leaf tea by the Wuhan MetWare Biotechnology Co., Ltd. via widely-targeted metabolome analyses (www.metware.cn). Standard procedures (Yuan et al., 2018) were followed for extraction, metabolite identification, and quantification. Briefly, after freeze drying sample, 70% methanol internal standard was added at a 30:1 ratio. After centrifugation, the supernatant was removed and stored in an injection vial for liquid chromatography-tandem mass spectrometry (LC-MS/MS). The ultra-performance liquid chromatography coupled with electrospray tandem mass spectrometry (UPLC-ESI-MS/MS; ExionLC™ AD, Sciex, Darmstadt, Germany) and the tandem mass spectrometry system (Sciex, Darmstadt, Germany) were used to analyze the metabolites of *A. sinensis* leaf tea extracts. Metabolites were quantified using the LC-ESI-Q TRAP-MS/MS (4000Q TRAP, ABI, United States) for multiple reaction monitoring (MRM).

The detected metabolites were classified into Class I and Class II based on the number of each metabolite, and their proportion in the total substance was analyzed by Microsoft Excel software (Version Microsoft 365, Microsoft Corporation, United States). The relative content of the primary metabolite was determined based on the peak map area of Class I. Then, the ratio of the relative content of each primary substance to the total content was also screened by Microsoft Excel software (Version Microsoft 365, Microsoft Corporation, United States).

### 2.9 Prediction of target genes of flavonoids in *A. sinensis* leaf tea and construction of the network

The Traditional Chinese Medicine Systems Pharmacology Database and Analysis Platform (https://old.tcmsp-e.com/tcmsp.php) was used to predict the target genes corresponding to the flavonoid metabolites. The compound-target network was generated using Cytoscape 3.9.1 based on transcriptomic and metabolomics data (Li et al., 2022).

### 2.10 Molecular docking

The structure of the metabolites (quercetin and eupatilin) was obtained from the PubChem database, and the SWISS-MODEL platform (https://swissmodel.expasy.org/) was used to generate the 3D structure of the target protein (Il1b and Cd40). Finally, AutoDock Tools (Version 1.5.7) was used for molecular docking, and as a calculation method, molecular docking was used to predict receptor-ligand binding modes, and PyMol 4.6.0 was used to visualize the docking results (Morris et al., 2009; Seeliger and de Groot, 2010).

### 2.11 Statistical analysis

The normality of the data was assessed through the application of the Kolmogorov–Smirnov test. In the event that the data exhibited a normal distribution, One-way ANOVA was employed, with adjustments made for multiple comparisons using Tukey’s test. Conversely, if the data did not meet the assumptions of parametric analysis, the Kruskal–Wallis test was utilized, with corrections for multiple comparisons made using Dunn–Sidak. The LC_50_ of *A. sinensis* leaf tea on zebrafish and the mortality rate of zebrafish larvae were determined using the Probit (assumes normal distribution) function in GraphPad 8.0 software. Additionally, Student’s t-test with Origin 2021 software (OriginLab, Northampton, Massachusetts, United States) was used for comparing two-group data. A significance level of *p* < 0.05 was considered statistically significant for all tests.

## 3 Results

### 3.1 Semi-lethal concentration of *A. sinensis* leaf tea on zebrafish

**FIGURE 1:**
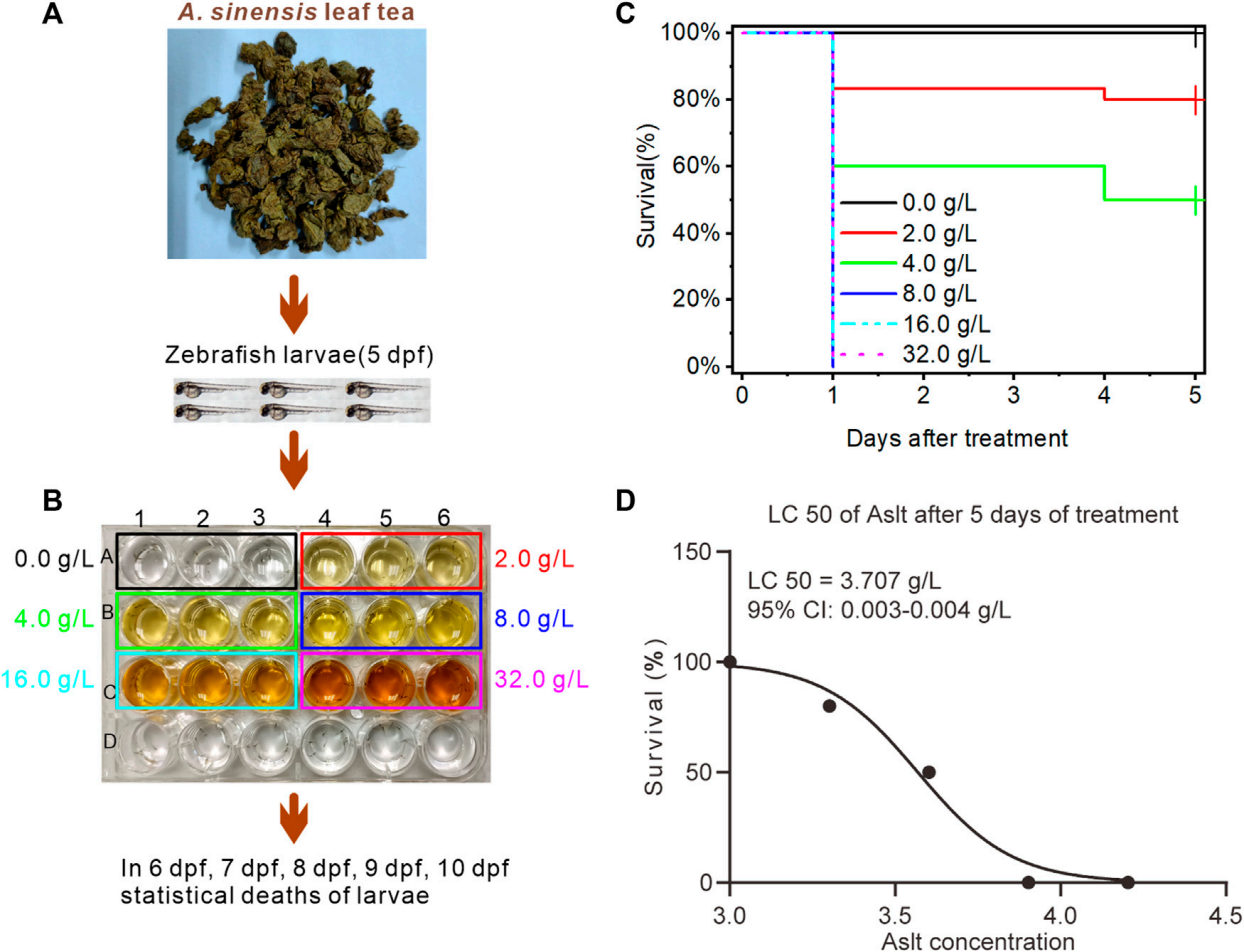
Effect of *Aquilaria sinensis* (Lour.) Spreng leaf tea extract on zebrafish larvae. **(A)** Treatment of zebrafish larvae at 5 dpf with *A. sinensis* leaf tea extract. **(B)** Zebrafish larvae exposed to six concentrations: 0.0 g/L (control), 2.0 g/L, 4.0 g/L, 8.0 g/L, 16.0 g/L, and 32.0 g/L (*n* = 90). The number of dead larvae was counted between 6 and 10 dpf. **(C)** The survival ratio of zebrafish larvae was calculated and plotted as Kaplan-Meier curves (*n* = 90). **(D)** LC_50_ of *A. sinensis* leaf extract (*n* = 90). Here, Aslt represents the *A. sinensis* leaf extract.

First, to determine the semi-lethal concentration (LC_50_) of *A. sinensis* leaf tea, 5 dpf zebrafish larvae were treated with six different concentrations (0.0 as a control, 2.0, 4.0, 8.0, 16.0, and 32.0 g/L; Figures 1A, B), and the number of dead larvae was recorded daily for 5 days after treatment (from 6 to 10 dpf). The survival curve showed that all the larvae treated at 5 dpf with *A. sinensis* leaf tea at 8.0, 16.0, and 32.0 g/L concentrations died at 6 dpf (the first day after treatment), while a few (16.67% and 40%) larvae treated with 2.0 and 4.0 g/L died. No new dead larvae were found in all the concentrations on 7 and 8 dpf (days 2–3 after treatment). At 9 dpf (the 4^th^ day after treatment), more larvae (20% and 50%) treated with 2.0 g/L and 4.0 g/L died, while no new deaths were observed in the other concentrations. No new larval deaths were recorded in any treatment groups on the 5th day after treatment (10 dpf) (Figure 1C). Further analysis found that on the 4^th^ day after treatment, the LC_50_ of the *A. sinensis* leaf tea on zebrafish larvae was 3.707 g/L, with a 0.003–0.004 g/L 95% confidence interval (Figure 1D).

### 3.2 *Aquilaria sinensis* leaf tea improves sleep in zebrafish with sleep disturbance

**FIGURE 2:**
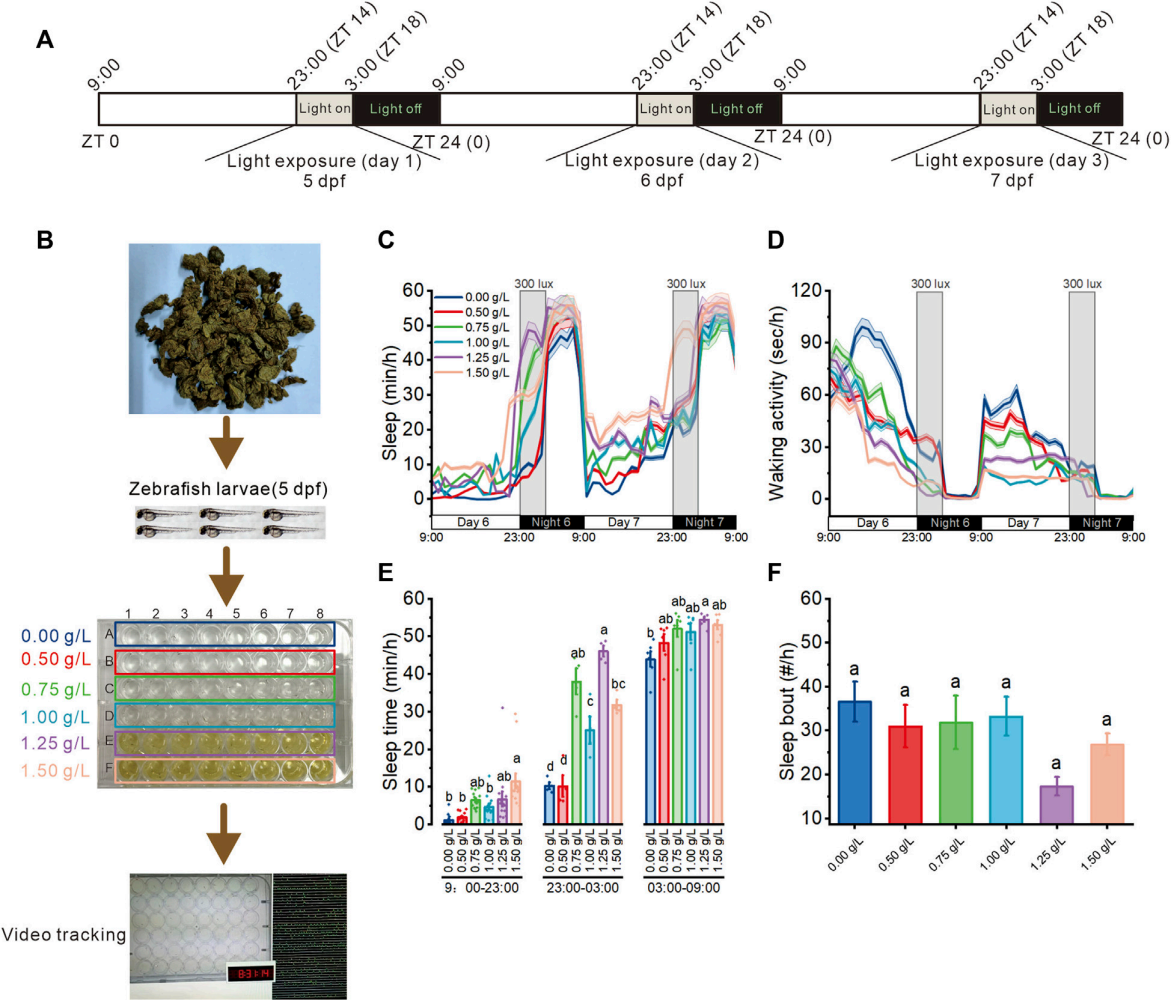
Effect of *Aquilaria sinensis* leaf tea on the sleep of zebrafish with sleep disturbance. **(A)** The schematic diagram shows sleep disturbance treatment. Sleep disturbance was performed for three consecutive days between 23:00 and 03:00 (ZT14-ZT18) from 5 dpf to 8 dpf. **(B)** Zebrafish larvae (5 dpf) were extract treated with six concentrations of *A. sinensis* leaf tea (0.00, 0.50, 0.75, 1.00, 1.25, and 1.50 g/L; *n* = 48) and placed in a behavior-tracking system. **(C,D)** Sleep and waking activity traces (±SEM) of zebrafish larvae treated with **(A)** *sinensis* leaf tea. The gray box indicates sleep disturbance time. (300 lux light on 23:00–03:00) (*n* = 48). **(E)** Quantification of total sleep across day time (9:00–23:00), sleep disturbance time (23:00–03:00), and night time (03:00–09:00) on 6 dpf (*n* = 48). **(F)** Sleep bout number between 23:00–03:00 (*n* = 48). (one-way ANOVA, Tukey’s *post hoc* test, Different lowercase letters indicate significant differences between various concentrations).

Further, to explore the effects of *A. sinensis* leaf tea extract on zebrafish with sleep disturbed (Figure 2A), 5 dpf larvae were exposed to six concentrations (0.00 as a control, 0.50, 0.75, 1.00, 1.25, 1.50 g/L, which less than half of LC_50_) of *A. sinensis* leaf tea extract (Figure 2B). Light exposure resulted in zebrafish sleep disturbance (Yokogawa et al., 2007). Only 1.50 g/L of *A. sinensis* leaf tea-treated larvae slept more compared to the other groups under day conditions, and no difference was observed among all groups at night time (without sleep disturbed time, 03:00–9:00). However, under sleep-disturbed conditions (23:00–03:00), the sleep time of zebrafish treated with 0.75, 1.00, 1.25, and 1.50 g/L of *A. sinensis* leaf tea was significantly increased compared with control (0.0 g/L) (Figures 2C–E) but no influence for the sleep bout (Figure 2F). These results suggest that *A. sinensis* leaf tea could improve the sleep of zebrafish under sleep disturbance conditions but could not change the sleep bout.

### 3.3 Transcriptome sequencing of *A. sinensis* leaf tea treated on sleep-disturbed zebrafish

**FIGURE 3:**
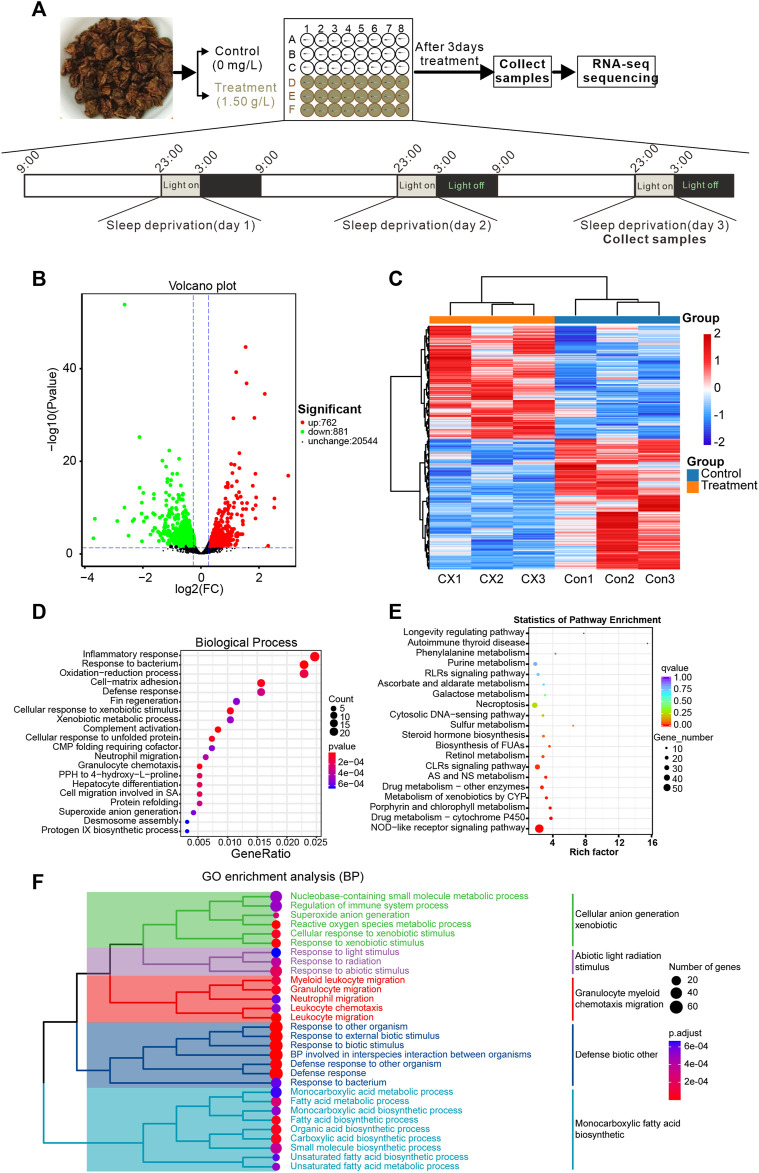
Transcriptome sequencing reveals changes in gene expression in zebrafish larvae exposed to *Aquilaria sinensis* leaf tea with sleep disturbance. **(A)** The schematic diagram shows the treatment and sample collection time of *A. sinensis* leaf tea-treated larvae under sleep disturbance conditions. The transcriptome of untreated larvae (0.00 g/L; Control) and larvae treated with 1.50 g/L tea (Treatment) and collected at 3:00 (ZT 18) on the third day of treatment. **(B)** The Volcano map displays the transcriptome sequencing results. **(C)** Heatmap of 1643 differentially expressed genes (*p*-value ≤0.05, log2FC ≥ 1.2). **(D)** GO enrichment analysis of the DEGs. **(E)** KEGG enrichment analysis showed that many genes were enriched in the NOD-like receptor. **(F)** The clustered heatmap of GO enrichment analysis, such as the regulation of the immune system process, myeloid leukocyte migration, granulocyte migration, neutrophil migration, leukocyte chemotaxis, and leukocyte migration.

This study found that 0.75–1.50 g/L concentrations of *A. sinensis* leaf tea significantly improved the sleep of zebrafish under sleep-disturbed conditions. Zebrafish treated with 1.50 g/L after 3 days of treatment were sequenced using 0.00 g/L as the control (Figure 3A). Transcriptome sequencing identified 1643 differentially expressed genes (DEG, *p*-value ≤0.05, log2FC ≥ 1.2), of which 762 were upregulated, and 881 were downregulated (Figures 3B, C). Further, GO enrichment analysis revealed that the DEGs were associated with inflammatory response (Figures 3D, F), and KEGG enrichment revealed that more DEGs were associated with the NOD-like receptor signaling pathway (Figure 3E). These observations suggest that *A. sinensis* leaf tea affects the inflammatory response of zebrafish under light-induced sleep disturbance conditions to improve sleep.

### 3.4 *Aquilaria sinensis* leaf tea influences immune response and sleep-related genes

**FIGURE 4:**
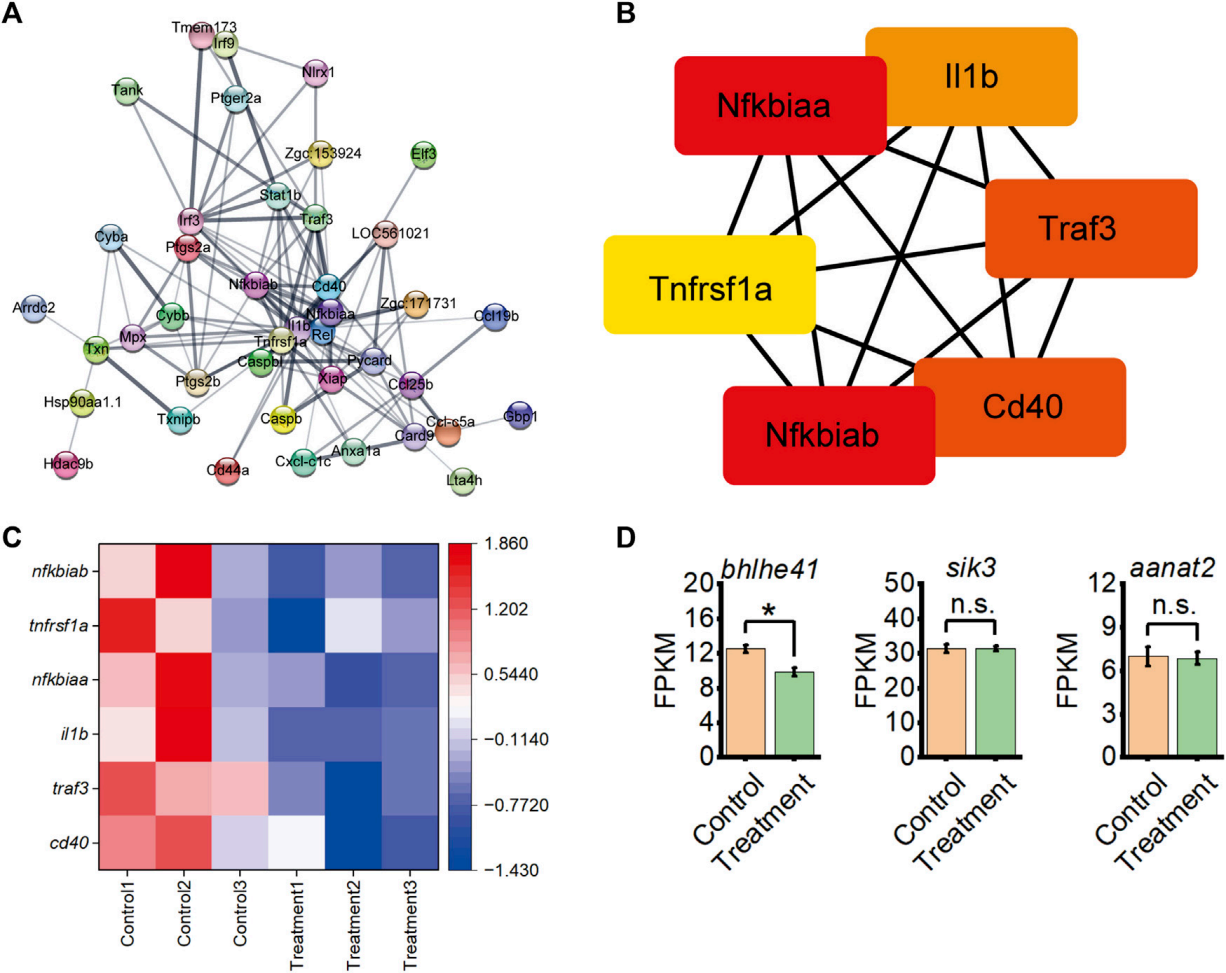
Inflammatory response interacts with the NOD-like receptor signaling pathway from DEG between control and 1.50 g/L *Aquilaria sinensis* leaf tea treatment groups and expression levels of sleep-related genes. **(A)** String analysis of DEGs associated with immune response. The thicker the lines between the proteins, the stronger the interaction. **(B)** Six immune-related genes were identified from String analysis. **(C)** Heatmap shows the expression of six immune-related genes under sleep disturbance conditions in DEGs. **(D)** FPKM of sleep-related genes between control and treatment group (one-way ANOVA, Tukey’s *post hoc* test. Data are shown as mean ± standard deviations. n.s. indicates no significance, * indicates significance at *p* < 0.05).

Further, the Cytoscape software performed a string protein-protein interaction analysis of DEGs enriched in the inflammatory response and NOD-like receptor signaling pathway (Figure 4A). This analysis identified six hub immune-related proteins from the inflammatory response and NOD-like receptor signaling pathway by protein-protein interaction of DEGs, including Nfkbiab, Tnfrsf1a, Nfkbiaa, Il1b, Traf3, and Cd40 (Figure 4B). A heatmap based on transcriptome data with the expression revealed the downregulation of genes encoding these six proteins in *A. sinensis* leaf tea zebrafish after treatment (Figure 4C). Further analysis of the expression levels of three sleep-related genes from transcriptome data, *bhlhe41*, *sik3*, and *aanat2*, revealed a downregulation of *bhlhe41* in zebrafish treated 3 days with *A. sinensis* leaf tea compared to the control under sleep disturbance conditions (Figure 4D). No significant changes were observed in *sik3* and *aanat2*, suggesting that *A. sinensis* leaf tea promotes the sleep of zebrafish under sleep-disturbed conditions by inhibiting the activity of *bhlhe41*.

### 3.5 *Aquilaria sinensis* leaf tea affects immune-related genes and expression

**FIGURE 5:**
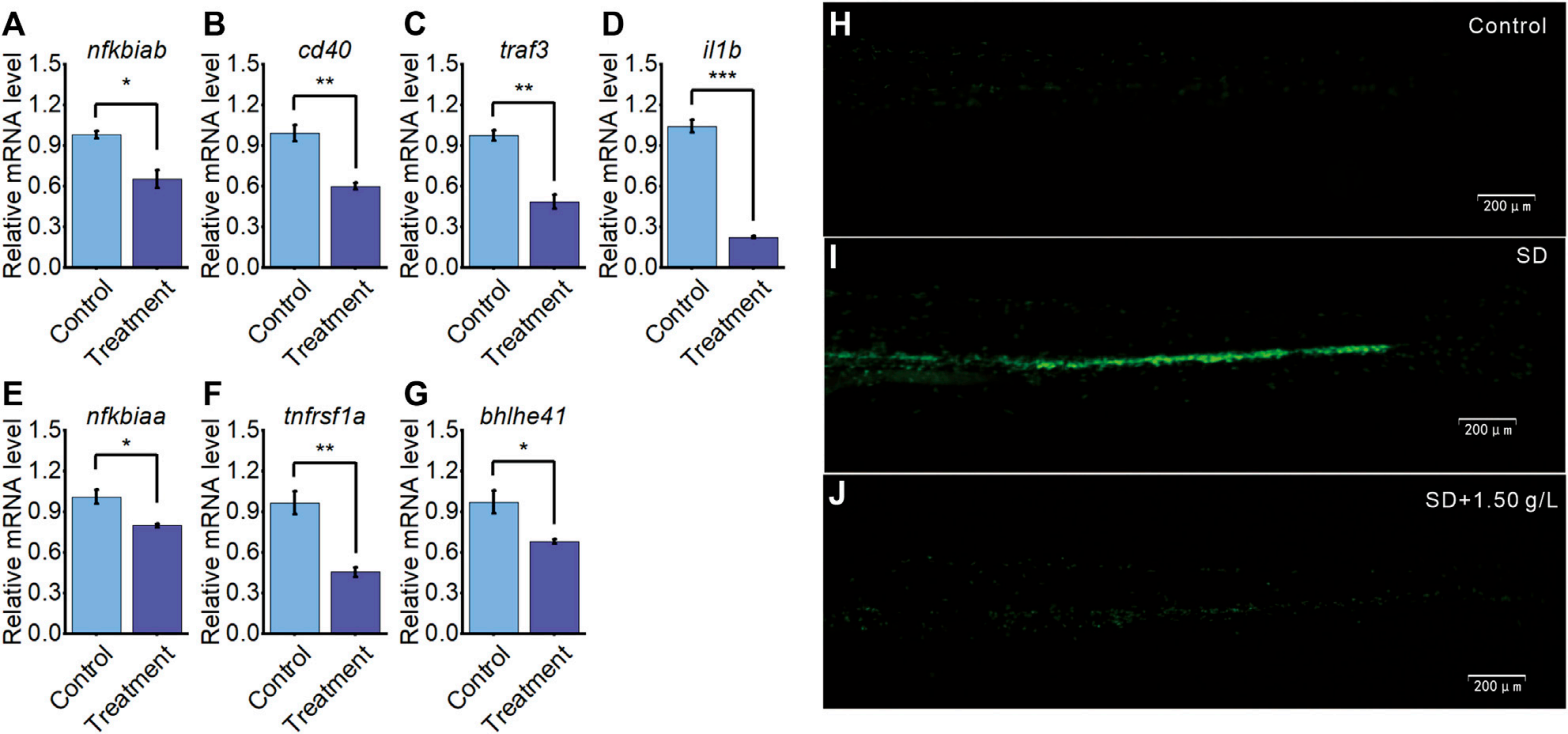
*A. sinensis* tea influences immune response and sleep gene on zebrafish sleep disturbed. **(A–G)** qRT-PCR validation of seven differentially expressed genes between the treatment group and the control group. **(H)** The neutrophil image of 5 dpf *Tg(mpo:GFP)* under normal condition (*n* = 30), **(I)** sleep disturbed condition (SD; *n* = 30), and **(J)** sleep disturbed condition with 1.50 g/L *A. sinensis* leaf tea treatment (*n* = 30) (one-way ANOVA, Tukey’s *post hoc* test. Data are shown as mean ± standard deviations. *: *p* < 0.05, **: *p* < 0.01, and ***: *p* < 0.001).

Transcriptome sequencing identified that the expression levels of six immune-related genes and one sleep-related gene were significantly downregulated in larvae treated with *A. sinensis* leaf tea during sleep disturbance. Subsequent qRT-PCR validated these results (Figures 5A–G), indicating the reliability and repeatability of the sequencing data.

To further validate the previous findings that *A. sinensis* leaf tea improves immune under zebrafish sleep disturbed, the 5dpf neutrophil-specific transgenic fish, *Tg(mpo:GFP)*, were divided into control group (no light exposed at 23:00–03:00), SD group (sleep disturbance, light exposed induced zebrafish sleep disturbed at 23:00–03:00) and SD + 1.50 g/L (sleep disturbance with 1.50 g/L of *A. sinensis* leaf tea). After 3 days, the SD group significantly induced neutrophil response compared with the control, and the SD + 1.50 g/L group improved neutrophils response compared with the SD group, suggesting that neutrophils migrated and aggregated significantly under sleep disturbance, *A. sinensis* leaf tea treated inhibit neutrophils aggregated through improves immune response (Figures 5H–J). These results further suggest that *A. sinensis* leaf tea improves the immune system of zebrafish under sleep disturbance conditions.

### 3.6 Chemical constituents of *A. sinensis* leaf tea and the target sites of flavonoids

**FIGURE 6:**
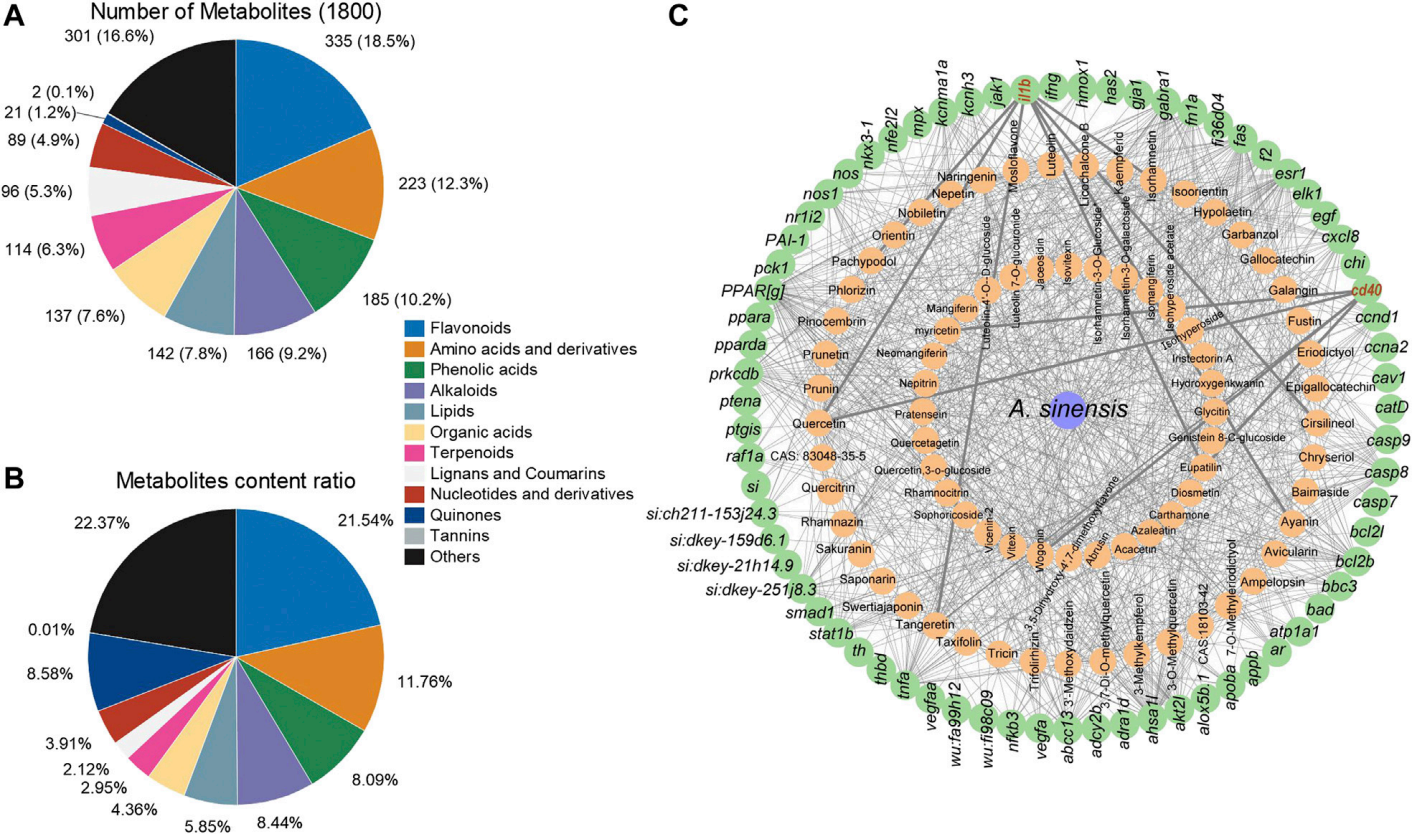
Analysis of metabolites in *Aquilaria sinensis* leaf tea and the gene targets of flavonoids. **(A)** Among 1800 metabolites were detected, 335 types of flavonoids accounted for the highest proportion. **(B)** The content of flavonoids obtained accounted for 21.54% in all metabolites, higher than other substances. **(C)** Flavonoid and target gene network (Green represents target sites and orange represents metabolites of *A. sinensis*).

The UPLC-MS/MS analysis of 1.50 g/L *A. sinensis* leaf tea identified 1800 chemical metabolites (Supplementary Table S2), of which flavonoids were the most abundant (335 metabolites) and accounted for 18.5% of the total components (Figure 6A). Additionally, the relative content of flavonoids was found to be the highest (21.54%; Figure 6B). Further analysis based on network pharmacology identified 70 target genes for the top 77 flavonoid metabolites in *A. sinensis* leaf tea. Interaction analysis showed that two out of all target genes screened from transcriptome data were the targets of flavonoids. Here, *il1b* was found to be the target gene of pachypodol, quercetin, isorhamnetin, cirsilineol, ayanin, tangeretin, and eupatilin, while *cd40* was the target gene of myricetin, quercetin, glycitin, eupatilin, and wogonin (Figure 6C). Among these, *il1b* and *cd40* were the common target genes of quercetin and eupatilin.

### 3.7 Molecular docking of two active components from *A. sinensis* leaf tea

**FIGURE 7:**
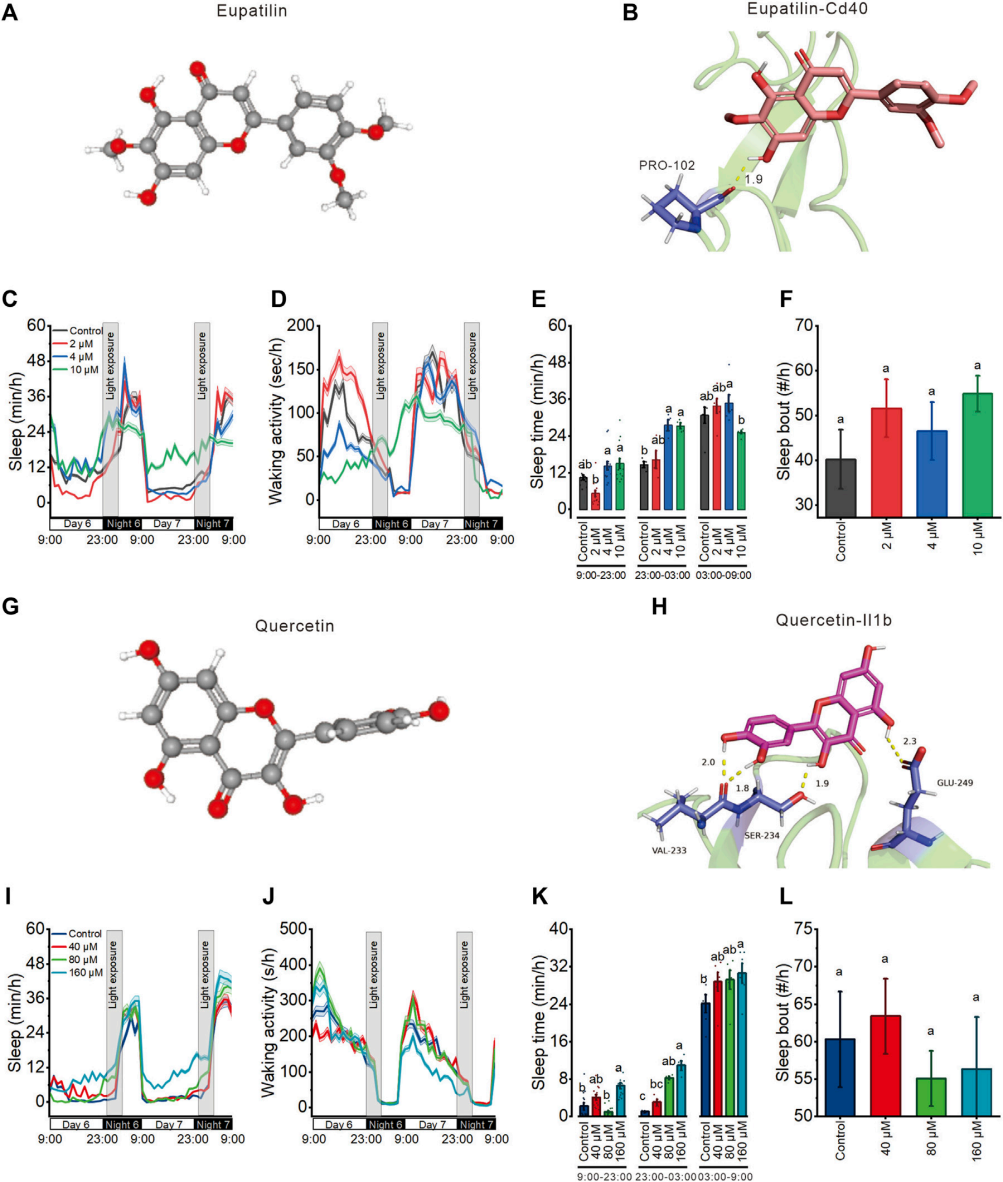
Eupatilin and quercetin, the potential active ingredients of *Aquilaria sinensis* leaf tea, improve the sleep of zebrafish with light-induced sleep disturbance. **(A)** 3D chemical structure of eupatilin based on PubChem database. **(B)** Molecular docking of eupatilin with Cd40. **(C–D)** Sleep and waking activity traces (±SEM) of zebrafish larvae treated with eupatilin. The gray box indicates sleep disturbance time (300 lux light on 23:00–03:00) (*n* = 48). **(E)** Quantification of total sleep across day time (9:00–23:00), sleep disturbance time (23:00 to 03:00), and night without sleep disturbance time (03:00–09:00) on 6 dpf in eupatilin treated and control groups (*n* = 48). **(F)** Quantification of sleep bout on sleep disturbance time between control and eupatilin treated groups (*n* = 48). **(G)** 3D chemical structure of quercetin based on PubChem database. **(H)** Molecular docking of quercetin with Il1b. **(I–J)** Sleep and waking activity traces (±SEM) of zebrafish larvae treated with quercetin (*n* = 48). **(K)** Quantification of total sleep across day time (9:00–23:00), sleep disturbance time (23:00 to 03:00), and night without sleep disturbance time (03:00–09:00) in quercetin treated and control groups on 6 dpf (*n* = 48). **(L)** Quantification of sleep bout on sleep disturbance time between control and quercetin-treated groups (*n* = 48). Lowercase letters indicate significant differences (one-way ANOVA, Tukey’s *post hoc* test. Data are shown as mean ± standard deviations. *p* < 0.05).

Based on the UPLC-MS/MS and network results, we screened eupatilin and quercetin (Figures 7A, G) and performed molecular docking with Cd40 and Il1b proteins that these two proteins modulate sleep. Molecular docking showed that eupatilin formed one hydrogen bond with PRO-102 in Cd40 (Figure 7B), while quercetin formed four hydrogen bonds with VAL-233, SER-234, and GLU-249 in Il1b (Figure 7H).

### 3.8 Eupatilin and quercetin are the major components of *A. sinensis* leaf tea that improve sleep in zebrafish with light-induced sleep disturbance

To finally investigate the effects of eupatilin and quercetin on zebrafish sleep with light-induced sleep disturbance, we treated 5 dpf larvae with these two components at three concentrations before the light-induced sleep disturbance. 4 μM and 10 μM eupatilin respectively increased sleep time during sleep disturbance time but had no influence during both day time and night time (Figures 7C–E). 80 μM and 160 μM quercetin respectively increased sleep time during sleep disturbance time but had no influence during day time, and 160 μM quercetin increased sleep at night time (Figures 7I–K) Moreover, both eupatilin and quercetin treated groups larvae exhibited no significant change in sleep bout (Figures 7F, L). These results indicate that eupatilin and quercetin are the potential active ingredients of *A. sinensis* leaf tea that improve zebrafish sleep under light-induced sleep disturbance.

## 4 Discussion

*Aquilaria sinensis* leaves are rich in bioactive substances (Nie et al., 2009; Qi et al., 2009; Feng and Yang, 2011; Yu et al., 2013; Yang et al., 2018) with potential biological effects. Specific studies have demonstrated that *A. sinensis* leaves possess anti-inflammatory, sedative, antioxidant, antimicrobial, laxative, and hypnotic effects (Hara et al., 2008; Zhou et al., 2008; Kakino et al., 2010; Li; Li et al., 2021; Liu et al., 2021). The present study investigated the effects of *A. sinensis* leaf tea on the sleep of zebrafish under sleep-disturbed conditions. Preliminary experiments found 3.707 g/L as the LC_50_ of *A. sinensis* leaf tea on 5 dpf zebrafish, which indicates non-toxicity according to the dose classification of acute toxicity (Huang et al., 2021). Based on this observation, a control and five concentrations of *A. sinensis* leaf tea were used to investigate the effects on zebrafish with disturbed sleep. Our results showed that *A. sinensis* leaf tea significantly promoted the sleep of zebrafish under sleep-disturbed conditions.

Sleep disturbance causes “a systemic low-grade inflammation” which is characterized by the release of cytokines and chemokines (Hurtado-Alvarado et al., 2016). Besides, animal studies have demonstrated the role of cytokines in regulating sleep (Kubota et al., 2001a; Kubota et al., 2001b; Marshall and Born, 2002; Opp, 2005; Clinton et al., 2011; Besedovsky et al., 2019). The transcriptome analysis of the present study revealed that 762 differentially expressed genes were upregulated, and 881 genes were significantly downregulated in zebrafish after *A. sinensis* leaf tea treatment. Most of these DEGs were associated with inflammatory response and NOD-like receptor signaling pathway. The String functional protein association network also displayed close interactions with 41 proteins, including six immune-related proteins (Nfkbiab, Tnfrsf1a, Nfkbiaa, Il1b, Traf3, and Cd40). Nfkbiab and Nfkbiaa are members of the nuclear factor of kappa light polypeptide gene enhancer in the B-cells inhibitor family, belonging to the NF-κB signaling pathway. The NF-κB signaling pathway has long been considered a typical pro-inflammatory signaling pathway (Pugaz et al., 2013). Besides, Leukin 1β (*il1b*) is one of the major mediators of the innate immune response and many studies found that Il1b levels were increased in human and animal models under sleep disturbance (Dinges et al., 1995; Krueger et al., 1999; Hu et al., 2003; Frey et al., 2007; Irwin et al., 2015; Lee et al., 2018). Cd40 regulation sleep through modulates genes associated with sleep homeostasis (such as Homer1a, Early growth response 2) (Gast et al., 2013; Karrer et al., 2015). Thus, the study indicates that *A. sinensis* leaf tea may improve sleep by affecting the inflammatory response of zebrafish under sleep disturbance.

In humans, the immune system is greatly affected by lack of sleep, a sufficient amount of sleep is associated with a reduction in neutrophils in the blood, and a lack of sleep affects the population diversity and function of circulating neutrophils in healthy adults (Christoffersson et al., 2014). The present study used the neutrophil-specific transgenic zebrafish *Tg (mpo: GFP)* to explore whether *A. sinensis* leaf tea can decrease neutrophil levels under sleep disturbance and found that neutrophils migrated and aggregated significantly under sleep disturbance. However, no significant migration of neutrophils was observed in zebrafish after *A. sinensis* leaf tea treatment during sleep disturbance. These results support the hypothesis that *A. sinensis* leaf tea could improve the immune system of zebrafish under sleep disturbance by modulating the expression of immune genes and neutrophils levels.

Metabolite profiling identified 1800 bioactive metabolites in *A. sinensis* leaf tea, of which flavonoids were the most abundant. Flavonoids are known to improve the immune system (Martinez et al., 2019). Min et al. also found that flavonoids constitute most of the ingredients in *A. sinensis* leaf extracts; however, using the UPLC-MS/MS method, they only identified 418 bioactive metabolites (Min et al., 2023). Hence, the present work identified more active ingredients of *A. sinensis* leaf tea using the same method. Further network and molecular docking analyses revealed that Il1b binds to quercetin and Cd40 to eupatilin. Research has shown that quercetin and quercetin 3-O-glucuronide regulate the sleep-wake cycle by activating the GABA(A) receptor and improving sleep duration and quality of insomnia mice (Kambe et al., 2010; Kim et al., 2021). Eupatilin has been shown to inhibit pancreatic cancer cells, the growth of kidney cancer cells, and the metastasis of prostate cancer cells while improving the immune system (Zhong et al., 2019; Serttas et al., 2021; Park and Kim, 2022). Our study first reports that eupatilin may improve sleep of a sleep-disturbed zebrafish, but it requires further investigation. Moreover, behavioral experiments showed that quercetin and eupatilin significantly improved sleep in zebrafish with light-induced sleep disturbance. Based on these findings, we speculate that quercetin and eupatilin play a crucial role in improving sleep in sleep-disturbance zebrafish; however, this hypothesis needs to be confirmed by further animal experiments.

## 5 Conclusion

Our study proved that *A. sinensis* leaf tea significantly improves the sleep quality of zebrafish with sleep disturbance due to light exposure. Transcriptome sequencing, qRT-PCR, and neutrophil transgenic fish imaging revealed that *A. sinensis* leaf tea promotes sleep through an improved immune response. Eupatilin and quercetin were identified as the potential active ingredients in *A. sinensis* leaf tea responsible for this effect. However, it requires further investigation and the development of natural products from leaves containing these two metabolites.

In summary, this study demonstrated that *A. sinensis* leaf tea promotes sleep and improves the immunity of zebrafish under light-induced sleep disturbance. These findings present a potential avenue for enhancing the sleep of individuals with sleep disturbance.

## Data Availability

The data presented in the study are deposited in the NCBI repository, accession number PRJNA1025141.
